# Percutaneous treatment of multivalvular heart disease following previous sternotomy

**DOI:** 10.1093/ehjcr/ytaf317

**Published:** 2025-07-02

**Authors:** Denizhan Ozdemir, Hasan Jilaihawi, Moody Makar, Raj Makkar

**Affiliations:** Division of Cardiology, Cedars-Sinai Medical Center, Smidt Heart Institute, 8700 Beverly Boulevard, Los Angeles, CA 90048, USA; Division of Cardiology, Cedars-Sinai Medical Center, Smidt Heart Institute, 8700 Beverly Boulevard, Los Angeles, CA 90048, USA; Division of Cardiology, Cedars-Sinai Medical Center, Smidt Heart Institute, 8700 Beverly Boulevard, Los Angeles, CA 90048, USA; Division of Cardiology, Cedars-Sinai Medical Center, Smidt Heart Institute, 8700 Beverly Boulevard, Los Angeles, CA 90048, USA

An 82-year-old patient with a long-standing history of multivalvular heart disease first underwent surgical mitral valve replacement (MVR) 14 years ago with a 27 mm Mosaic porcine bioprosthesis for infective endocarditis. Seven years later, he developed a mitral paravalvular leak, which was closed percutaneously; however, the procedure was complicated by ventricular fibrillation, necessitating an implantable cardioverter-defibrillator.

Six years ago, due to progressive severe aortic stenosis, he received a 29 mm SAPIEN 3 transcatheter aortic valve replacement (TAVR). One year following TAVR, he presented with severe symptomatic tricuspid regurgitation (TR) and underwent a transcatheter tricuspid edge-to-edge repair (T-TEER) using two TriClip devices in anteroseptal and anteroposterior positions, which initially improved his symptoms. However, 3 years later, imaging revealed single leaflet device attachment of the anteroseptal clip, resulting in the recurrence of severe TR.

A second T-TEER attempt was unsuccessful because of a wide coaptation gap, and he was deemed ineligible for a transcatheter tricuspid valve replacement (TTVR) under the TRISCEND II study. Despite maximal medical therapy, his TR progressed to a massive degree, causing repeated heart failure admissions. Following the commercial approval of the EVOQUE TTVR system, the patient underwent a successful implantation, considering his persistently massive TR, previous sternotomy, high surgical risk, and frailty.

Over time, the bioprosthetic mitral valve began to deteriorate, causing mitral stenosis, and the patient underwent a valve-in-valve transcatheter mitral valve replacement (TMVR) using a 26 mm SAPIEN device.

Since his initial paravalvular leak closure, he has successfully undergone TAVR, TTVR-in-TriClip, and TMVR-in-valve procedures without complications (*Figure 1*). He has been maintained on warfarin for atrial fibrillation and continues to enjoy a satisfactory quality of life. There is a lack of randomized data to guide treatment in multivalvular disease.^1–3^ In this context, multidisciplinary heart team discussions, imaging, and institutional expertise play critical roles in managing these patients.^1^

**Figure 1:**
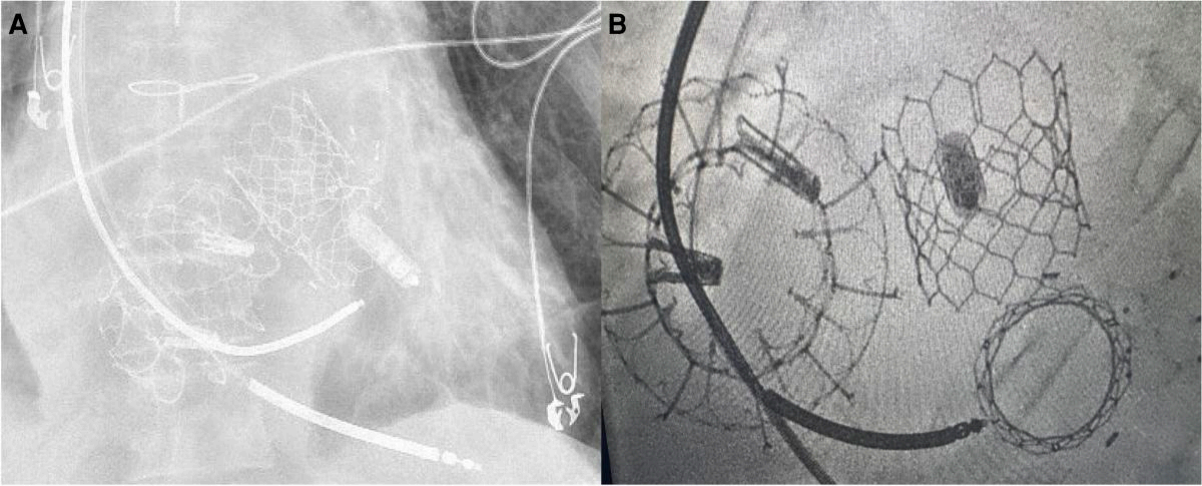
(*A*) Chest X-ray image of Sapien TAVR, EVOQUE TTVR-in-Triclip, and Sapien TMVR-in-valve; (*B*) fluoroscopic image of Sapien TAVR, EVOQUE TTVR-in-Triclip, and Sapien TMVR-in-valve with prior paravalvular leak occluders for paravalvular surgical valve leak.

## Data Availability

The data underlying this article are available in the article and in its online supplementary material.
